# Ionic Inter-Particle Complexation Effect on the Performance of Waterborne Coatings

**DOI:** 10.3390/polym13183098

**Published:** 2021-09-14

**Authors:** Maialen Argaiz, Fernando Ruipérez, Miren Aguirre, Radmila Tomovska

**Affiliations:** 1 POLYMAT and Departamento de Química Aplicada, Facultad de Ciencias Químicas, University of the Basque Country UPV/EHU, Joxe Mari Korta Zentroa, Tolosa Hiribidea, 72, 20018 Donostia-San Sebastián, Spain; maialen.argaiz@polymat.eu (M.A.); miren.aguirre@ehu.eus (M.A.); 2 POLYMAT and Physical Chemistry Department, Faculty of Pharmacy, University of the Basque Country UPV/EHU, 01006 Vitoria-Gasteiz, Spain; fernando.ruiperez@ehu.eus; 3 Ikerbasque, Basque Foundation for Science, Maria Diaz de Haro 3, 48013 Bilbao, Spain

**Keywords:** waterborne coating, emulsion polymerization, meth(acrylic) latex, ionic complexation, inter-particle complexation, sodium styrene sulfonate, 2-(dimethylamino)ethyl methacrylate

## Abstract

The performance of waterborne (meth)acrylic coatings is critically affected by the film formation process, in which the individual polymer particles must join to form a continuous film. Consequently, the waterborne polymers present lower performance than their solvent-borne counter-polymers. To decrease this effect, in this work, ionic complexation between oppositely charged polymer particles was introduced and its effect on the performance of waterborne polymer films was studied. The (meth)acrylic particles were charged by the addition of a small amount of ionic monomers, such as sodium styrene sulfonate and 2-(dimethylamino)ethyl methacrylate. Density functional theory calculations showed that the interaction between the selected main charges of the respective functional monomers (sulfonate–amine) is favored against the interactions with their counter ions (sulfonate–Na and amine–H). To induce ionic complexation, the oppositely charged latexes were blended, either based on the same number of charges or the same number of particles. The performance of the ionic complexed coatings was determined by means of tensile tests and water uptake measurements. The ionic complexed films were compared with reference films obtained at pH at which the cationic charges were in neutral form. The mechanical resistance was raised slightly by ionic bonding between particles, producing much more flexible films, whereas the water penetration within the polymeric films was considerably hindered. By exploring the process of polymer chains interdiffusion using Fluorescence Resonance Energy Transfer (FRET) analysis, it was found that the ionic complexation was established between the particles, which reduced significantly the interdiffusion process of polymer chains. The presented ionic complexes of sulfonate–amine functionalized particles open a promising approach for reinforcing waterborne coatings.

## 1. Introduction

The global market of polymer-based paints and coatings is projected to grow substantially over the period of 2019–2025, owing to the worldwide increasing demand by many different sectors, such as the construction and automotive sectors, aerospace, mining and packaging production industries [1,2]. However, many industrial polymer production processes relay still on the use of organic solvents, which contributes to increase emissions of volatile organic compounds (VOC). Along with environmental concerns, the stringent environmental standards for VOC’s emission have been a driving force for replacing processes that use solvents by cleaner water-based ones [3,4,5]. Nevertheless, the water-based polymers still present lower mechanical performance than their solvent-based counter products [6,7]. While from the polymer solution, the films formed after solvent evaporation are perfectly continuous, the film formation process from polymer aqueous dispersions (latexes) is much more complex. The polymer chains are enclosed within submicron particles that during the water evaporation have to join through polymer chains’ interdiffusion. The worst mechanical performance and considerable water sensitivity of waterborne films is directly related to the film formation process [8,9] and is usually responsible for the lower performance of the films, which slow down the replacement of the solvent-borne coatings by the waterborne ones on the market.

To improve the performance of the waterborne coatings, several approaches have been studied, either based on enhancing the diffusion of hard polymer chains or addition of functionalities to soft polymer chains. The diffusion of polymer chains could be enhanced by the addition of plasticizers, a strategy extensively used in industry [10,11,12,13]. The incorporation of coalescence agents initially softens the polymer particles to form a coherent film. Typically, during the film formation process, water together with these compounds is evaporated, giving rise to hard and non-tacky materials [10,11], which increases the VOC emissions. The use of water as hydroplasticizer has been reported for certain polymer types [14,15] as a strategy to design low VOC latexes, without compromising the mechanical resistance of the polymer films. Alternatively, the chains diffusivity can be improved by blending low-T_g_- and high-T_g_-polymer latexes [16,17] or developing multiphase systems that combine hard and soft polymer domains within the same polymer particle [18]. In both cases, while hard polymer provides mechanical strength, soft fraction allows proper polymer chain mobility to complete the film formation process. Likewise, in some works polymer nanocomposites are combined with inorganic fillers to obtain improvements in mechanical strength of the final product [19].

Reinforcement of soft polymer films by inducing inter-particle chemical and physical bonding is an alternative approach towards improved performance of waterborne coatings [20,21,22]. Although chemical crosslinking is widely used in industry, it presents some drawbacks, as the use of chemicals (as isocyanates, aziridines and carbodiimmides) in the self-crosslinking reactions is under scrutiny due to their toxicity [13,20,23]. One alternative approach is the formation of a physical network through hydrogen bonding or ionic interactions established between the particles during the film formation [21,22,24]. As shown by Jimenez et al., addition of tannic acid aqueous solution to acrylic latex containing pyrrolidone groups led to a physical network formed by hydrogen bonding [25]. Even though polymer films with improved mechanical properties were obtained, the H- bonding network could not hinder water absorption by the polymer films.

Several works have been reported in recent years in which the efficiency of electrostatic interactions have been demonstrated for different applications, such as drug delivery and tissue engineering [25,26,27]. Formation of ionic supramolecular network between ammonium and carboxylic acid moieties was reported by addition of adipic and citric acid in waterborne free-isocyanate polyurethane (WHPH) dispersions [28]. The resulting materials presented enhanced mechanical properties and exhibited self-healing abilities. Furthermore, the formation of a percolating network between methacrylic acid containing latex (40% solid content, s.c.) and ZnO nanoparticles was reported, resulting in tunable mechanical properties [29]. Likewise, it was demonstrated that the addition of ZnO/KOH inorganic material into a latex containing methacrylic acid groups (45% s.c.) led to the formation of an ionic network that improved the elastic modulus of the material [30].

The above works inspired us to study the ionic inter-particle complexation of waterborne polymer particles and their effect on the final performance of the polymer film. In this line, Tiggelman et al. blended two oppositely charged 30% solids content waterborne latexes made of methyl methacrylate (MMA) and butyl acrylate (BA), using small amount of acrylic acid (AA) or 2-(dimethylamino) ethyl methacrylate (DMAEMA) as anionic and cationic functional monomers [31]. It was shown that, by a simple acid-base proton transfer, an ionic crosslinking was induced, which influenced the film properties. Thus, by increasing the ionic content, the adhesion energy of the films to the substrates decreased, whereas their water sensitivity increased. However, the ionic complexation process was not studied, neither the effect of important parameters such as pH, surface charge density or particle size. The reported increased water sensitivity indicated presence of a higher number of free, non-complexed ions.

To shed a bit of light on the ionic complexation process in oppositely charged latex blends and the ionic bonding effect on the performance of the resulting films, in this work, oppositely charged polymer particles were synthesized by a two-step emulsion polymerization process of the basic coating formulation made of MMA and BA in 50/50 wt. ratio. NaSS and DMAEMA ionic functional monomers were used in small amounts (1–3% with respect to MMA/BA weight) to produce charged particles. The functional monomers were selected to provide a wide pH range between both pKa values, in which the ionic bonding can be raised. The possibility of ionic bonds formation between the selected opposite ionic monomers was studied by means of Density Functional Theory (DFT) calculations. The blends of the oppositely charged latex were prepared either on the base of similar surface charge density or a similar number of particles. To get an insight on the effect of ionic crosslinking onto the interdiffusion ability of the polymer chains during film formation process, direct energy transfer (ET) was monitored in films prepared from polymer particles labeled with (9-phenanthryl) methyl methacrylate (Phe-MMA) as the donor and 1-(4-nitrophenyl)-2-pyrrolidinmentyl]-acrylate (NNP-A) [32] as the acceptor by FRET technique. This work demonstrates that, by the simple blending of oppositely charged polymer latexes, the performance of the resulting coating films can be significantly enhanced, especially the water sensitivity and flexibility.

## 2. Materials and Methods

### 2.1. Materials

Technical grade monomers n-butyl acrylate (BA, Quimidroga, Barcelona, Spain), methyl methacrylate (MMA, Quimidroga, Barcelona, Spain), sodium p-styrene sulfonate (NaSS, Sigma-Aldrich, Madrid, Spain), 2-(dimethylamino)ethyl methacrylate (DMAEMA, Sigma-Aldrich, Madrid, Spain) were used as monomers. Disponil AFX 2075 (BASF, Ludwigshafen, Germany) non-ionic emulsifier was used as received. The initiator tert-butyl hydroperoxide (TBHP, 70 wt% aqueous solution, Luperox Sigma-Aldrich, Madrid, Spain) and ascorbic acid (AsAc, purity ≥ 99%, Sigma-Aldrich, Madrid, Spain) were also used as received. Hydroquinone (Fluka, Madrid, Spain) was used to stop the polymerization reaction in the samples withdrawn from the reactor. Technical grade tetrahydrofuran (THF, Scharlab, Madrid, Spain) and HPLC grade THF (Scharlab, Madrid, Spain) were used for Soxhlet extraction and Size Exclusion Chromatography measurements, respectively. Hydrochloric acid (HCl, 37%, Sigma-Aldrich) was used to decrease the pH of cationically charge latex for later titration analysis and sodium hydroxide (NaOH, Sigma-Aldrich, Madrid, Spain) was employed for the titration of both latexes. Ammonium hydroxide solution (28% NH_3_ in water, Sigma-Aldrich, Madrid, Spain), and NaOH (Sigma-Aldrich, Madrid, Spain) were all used for buffer preparations. (9-phenanthryl) methyl methacrylate (Phe-MMA, Toronto Research Chemicals, Toronto, Canada) and [1-(4-nitrophenyl)-2-pyrrolidinmentyl]-acrylate (NNP-A, Sigma-Aldrich, Madrid, Spain) [32] were employed as donor and acceptor pair to carry out FRET analysis.

### 2.2. Computational Details

All geometry optimizations were carried out within density functional theory (DFT) using the M062X functional [33] combined with the 6-31+G(d,p) basis set [34]. To confirm that the optimized structures were minima or transition states on the potential energy surfaces, frequency calculations were performed at the same level of theory. These frequencies were then used to evaluate the zero-point vibrational energy (ZPVE) and the thermal corrections, at T = 298.15 K, in the harmonic oscillator approximation. Single-point calculations using the 6-311++G(2df,2p) basis set [35] were performed on the optimized structures in order to refine the electronic energy. All the calculations were carried out with the Gaussian 16 suite of programs [36].

### 2.3. Synthesis of Waterborne Polymer Latexes

**Figure 1:**
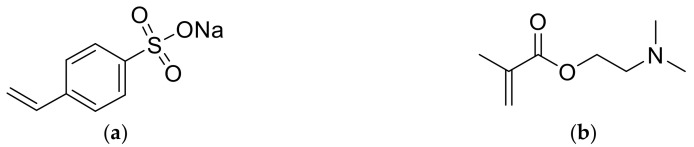
Chemical structure of (**a**) NaSS anionic monomer and (**b**) DMAEMA cationic monomer.

Ionically charged waterborne polymer latexes were synthesized by a two-step seeded semibatch emulsion polymerization process. A typical acrylic formulation, 50/50 MMA/BA was used, and two different functional monomers were chosen to give the ionic character to the latexes. NaSS (Figure 1a) was used as the anionic functional monomer, which was incorporated onto MMA/BA polymer particles following the synthesis procedures reported by Bilgin et al. [37]. DMAEMA was selected as cationic monomer (Figure 1b) due to its pH dependency, which might help to control the amine protonated state.

**Scheme 1:**
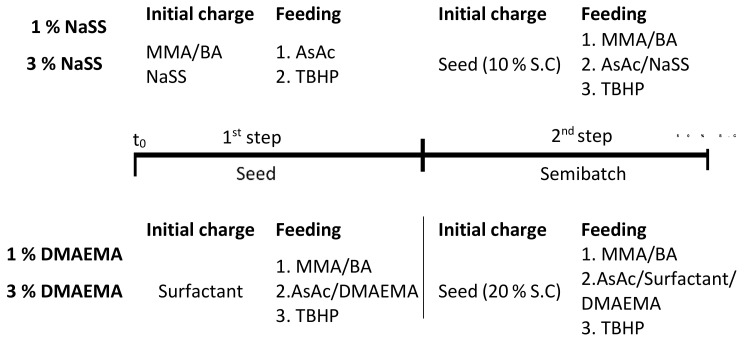
Schematic representation of the synthesis process for anionically and cationically charged latexes.

Initially, a seed with MMA/BA (50/50 wt%) at 10% s.c. for anionically charged system and 20% for cationic one was prepared. A schematic representation of these processes is illustrated in Scheme 1. Formulations for the seed synthesis of anionically and cationically charged latexes are shown in Table S1 in Supporting Information.

In a second step, the seed was grown by semibatch emulsion copolymerization. The ratio between the main monomers, MMA and BA, was maintained 1/1 by weight. The functional monomers content (NaSS and DMAEMA) was varied between 1 and 3% with respect to the main monomers (MMA and BA) amount in weight (wbm%). The synthesis of this second part was carried out as follows. The reactor was loaded with the desired amount of seed (39% for anionic and 44% for cationic latex) and the temperature was increased to 70 °C and 60 °C for anionic and cationic latex, respectively. Upon achieving the desired temperature, initiator redox couple aqueous solution (0.5 wbm% and 1 wbm% for anionic and cationic latexes, respectively), a preemulsion containing MMA, BA, F.M and the surfactant (4 wbm%) for the cationic system were fed in the three streams. The final solids content for each latex was 50%. After the feeding period (210 min and 240 min for anionic and cationic dispersion, respectively), the system was allowed to react for one more hour. Formulations are shown in Table S2 in the Supporting Information and a schematic view of NaSS and DMAEMA containing latex synthesis processes is illustrated in Scheme 1.

In order to carry out FRET analysis, a fluorescent dye donor and acceptor molecule have to be covalently linked into the polymer backbone [38,39]. Phe-MMA donor molecule was added to MMA/BA monomers preemulsion for anionically charged latex (1% NaSS-FRET), while NNP-A acceptor dye molecule to MMA/BA monomers preemulsion for cationically charged latex (1% DMAEMA-FRET). Both latexes were covalently labeled with 1 mol% of fluorescent dye based on major monomers (MMA/BA). For the sake of comparison, the latexes were synthesized following the same procedure as the one described in the previous section, but this time reactions were performed in 100 mL reactors. For more details, check the Supporting Information.

### 2.4. Latex Characterization

Monomer conversion was studied gravimetrically, and particle sizes were measured by dynamic light scattering (DLS) using a Zetasizer Nano Z (Malvern instruments, Malvern, UK). Samples were prepared by diluting a fraction of latex with deionized water. The equipment was operating at 25 °C and the reported values were the Z-average of three repeated measurements.

The gel fraction, which is defined as the insoluble fraction of polymer in THF, was measured by Soxhlet extraction and calculated as shown elsewhere [40]. Molar mass of the soluble fraction (obtained from Soxhlet extraction) was determined by Size Exclusion Chromatography/Gel Permeation Chromatography (SEC/GPC) Samples were dried at room temperature, re-dissolved in THF and filtered using polyamide 0.45 µm filter before injected into the GPC, which consisted of a pump (Schimadzu LC-20AD), three columns in series (Styragel HR2, HR4 and HR6) and a refractive index detector (Waters 2410). Chromatographs were obtained at 35 °C using a THF flow rate of 1 mL/min. The molar masses were related to polystyrene standards.

The surface charge density of the latex was determined by titration of the dialyzed latexes. These latexes were diluted to 2.5 wt% solids content and dialyzed against deionized water using Spectra-Por^®^4 membranes (Mw cut-off 12,000–14,000 Da) until the conductivity of the dialysate was close to that of deionized water (2 µS/cm). For the anionically charged polymer latexes, the dialyzed latexes were passed through a Dowex Marathon MSC cation exchange resin in order to substitute Na^+^ of the sulfonate groups by titratable H^+^. The pH of the latex containing DMAEMA functional monomer was reduced in order to ensure the ionic state of the species [41]. Both latexes were titrated conductimetrically using Metrohm 718 stat titrino equipment (Bangkok, Thailand) against 0.015 M NaOH solution. The surface charge density (σ) was calculated according to Equation (1) [42,43]:

(1) $$\sigma =\frac{Fn\rho R}{3w}$$

where *F* is the Faraday constant, *n* is the number of moles of NaOH required to neutralize the functional groups of the latex, *ρ* is the polymer particle density, *R* is the radius of polymer particles and *w* is the solid fraction of the latex.

Water-soluble species were calculated by titration. The diluted latexes (12.5 wt% solid content) were centrifuged at 30,000 rpm for 3 h at 4 °C. For the anionic latex, the serum part was carefully taken with a syringe and pass through a Dowex Marathon MSC cation exchange resin in order to substitute Na^+^ of the sulfonate groups by titratable H^+^. In case of the cationic latex, the serum was taken, and the pH was decreased to ensure an ionic state of DMAEMA molecules. Both serums were titrated against 0.015 M NaOH.

### 2.5. Films Characterization

Films were first dried in silicon molds at 23 ± 2 °C and 55 ± 5% relative humidity.

The glass transition temperature (T_g_) of the polymers was determined using a differential scanning calorimeter, DSC (Q1000, TA Instruments) (Hüllhorst, Germany). The scanning cycles consisted of first cooling to −50 °C at 10 °C/min, then heating from −50 to 150 °C at 10 °C/min, cooling again from 100 to −50 °C at 10 °C/min, and then heating to 150 °C at a rate of 10 °C/min. The results from the second heating run from −50 to 100 °C will be presented herein.

Water contact angle measurements of each blend film were performed by placing 12 μL droplets of distilled water on the surface of the films, using a goniometer OCA 20 (Data Physics Instrument, Filderstadt, Germany) under controlled environment (23 ± 2 °C and 55 ± 5% humidity). The data presented are the average of 15 readings.

Tensile test measurements were performed in a Universal Testing Machine, (TA.HD plus Texture Analyzer) (Godalming, UK) under the same temperature and humidity conditions and applying a crosshead speed of 25 mm/min to an approximate 0.6 mm thick polymer film. At least five specimens per sample were tested and the average value is reported.

For the water uptake test, 0.6 mm thickness samples were prepared in silicon molds and dried at 23 ± 2 °C and 55 ± 5% relative humidity. Materials were tested upon immersion in water for 2 weeks. At some intervals, films were taken out of the vials and smoothly blotted with paper and weighted. Water uptake was calculated in relation to the initial weight. After water uptake test, films were dried at 62 °C for 5 days and the weight loss was reported. This value was normalized against the initial weight of the sample. Each material was tested twice, and the given value is an average.

Fluorescence Resonance Energy Transfer (FRET) was employed to determine the extent of interdiffusion at microscopic level using Fluoromax-4 spectrofluorometer (Horiba Jobin Yvon, Kyoto, Japan) equipped with a single photon counting controller (FluoroHub) and a pulsed diode light source NanoLED emiting at 300 nm. DAS6 Fluorescence decay analysis software was applied for analysis of time domain fluorescence lifetimes. For more details about the experimental procedure, see the Supporting Information.

### 2.6. Blends Preparation and Film Formation

**Figure 2:**
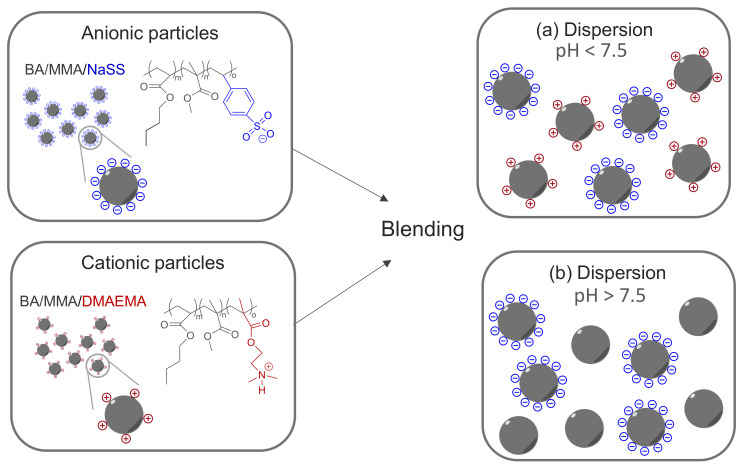
Schematic representation of the blends between NaSS and DMAEMA functionalized latex.

To minimize the effect of water-soluble compounds during the ionic interaction between the oppositely charged polymer particles, the latexes were dialyzed, eliminating the water-soluble species together with the non-ionic surfactant from the waterborne latex. Conductivity of water was followed until a value around 2 µS/cm was achieved, which corresponds to the deionized water conductivity. The main characteristic of NaSS specie is its relatively low pKa (around 1) [37], while pKa of poly(DMAEMA) is around 7.5 [44]. This means that when the pH is below 7.5 all the ionic species are in their cationic and anionic state favoring the formation of the ionic network between oppositely charged polymer particles, whereas when the pH is higher than 7.5, the DMAEMA molecules are in their molecular form avoiding the interaction between the oppositely charged ionic groups (Figure 2).

The blends were first prepared by mixing both latexes based on the equal number of opposite charges (Table 1). Blends were first performed between 1% and 3% ionic monomer containing latex (Blend C1-1 and Blend C3-3, respectively) to study the effect of density charge on the final performance of the films. As the incorporation of the NaSS into polymer particles is higher than incorporation of DMAEMA (these results are presented in Table 2), more amount of DMAEMA containing latex was added to the blends in order to obtain equal number of oppositely charge species. Furthermore, blends between 1% NaSS containing latex and 3% DMAEMA containing one (Blend C1-3) were also prepared since both latex present similar surface charge density values, 16 µC/cm^2^ and 19 µC/cm^2^, respectively, as shown in Table 2, and therefore, in this case clearer effect of ionic bonding was expected to be observed. A summary of the prepared blends is shown in Table 1.

The effect of the ionic network might be screened in case of Blend C1-1 and Blend C3-3 owing to the higher amount of cationic species added to the blend. Therefore, in order to increase the ionic bonding points of 1% and 3% systems, blends were also performed based on the equal number of opposite particles (Blend P1-1 and Blend P3-3, respectively). As 1% and 3% NaSS containing latex show higher particle size (Table 2) than 1% and 3% DMAEMA ones, greater amount of NaSS latex was added to these blends. Moreover, blends between 1% NaSS and 3% DMAEMA containing latex (Blend P1-3) were also employed to further investigate the effect of blending particles with similar density charge. A summary of these blends is presented in Table 1.

Blends were named as Blend C and Blend P indicating whether the blends were performed considering the surface charge density (Blend C) or the number of particles (Blend P). The numbers refer to the concentration of each latex used (NaSS containing latex-DMAEMA containing latex).

The blends prepared at pH > 7.5 will be used as the reference material due to the lack of ionic interaction at this pH. To control the pH of these blends, ammonium hydroxide (NH_4_OH) and sodium hydroxide (NaOH) solutions were used. The main difference between these two solutions is the volatility of NH_3_. Since NH_4_OH is a weak base, an equilibrium exists between the ammonium cation ($NH_{4}^{+}$) and the ammonia ($NH_{3}$) in the aqueous solution as presented in Equation (2) [39,45]:

(2) $$NH_{4}^{+}\left(aq.\right)+OH^{-}\left(aq.\right)↔\;NH_{3}\;\left(g\right)\;↑+H_{2}O\;\left(l\right)$$

As $NH_{3}\;$ is a volatile compound, and during the drying process, *NH*_3_ will evaporate, shifting the equilibrium to the right side and decreasing the concentration of $NH_{4}OH\;$ in the medium [39]. The effect of the NH_3_ evaporation leads to a drop in the pH and, therefore, the tertiary amine groups presented in the DMAEMA specie turned into a protonated state. On the contrary, sodium hydroxide is a non-volatile specie. This means that once NaOH solution (pH 11) is added to the blend, the pH of this solution will remain constant, around 9, allowing the preparation of reference films and ensuring no ionic interactions.

In all the cases, solutions were added to the cationically charged latexes while stirring. Once the pH of the DMAEMA containing latex was above 7.5, the anionically charged latex was added, forming the blend. Blends were mixed for 2 h before casting the films. The blends were cast into silicon molds and they were let drying for 7 days at 23 ± 2 °C and 55 ± 5% relative humidity.

### 2.7. Blends Preparation and Film Formation for Dye Latexes

Dye-labeled latexes were blended following the same methodology. Blends were performed employing 1% NaSS and 1% DMAEMA containing latex at different pHs (pH < 7.5 and pH > 7.5). Blending was prepared considering the two parameters mentioned before: surface charge density and number of particles. After mixing for 2 h, few drops of each blend were casted into glass substrates. Films were allowed to dry under standard conditions (23 ± 2 °C and 55 ± 5% relative humidity). The time at which both films appeared dry and transparent was taken as time zero (*t*_0_) for FRET experiments. The polymer interdifussion process was monitored following the evolution of the fluorescent decay profile *I_D_(t)*. The results are given in terms of the quantum efficiency energy transfer ($\Phi_{ET}$), which is related to the fraction of molecular mixing in a system of labeled particles and it is defined as [46,47,48]:

(3) $$\Phi_{ET}\;\left(t\right)=1-\frac{\int_{0}^{\infty }I_{D}\;\left(t\right)dt}{I_{D}^{0}\;\left(t\right)\;dt}$$

where $\int_{0}^{\infty }I_{D}\;\left(t\right)$ refers to the integrated area under the normalized decay profile and $I_{D}^{0}\;\left(t\right)$ is defined as the donor decay profile of the film containing donor fluorescence molecule ($\tau_{D}^{0}$). More details can be found in the Supporting Information.

## 3. Results and Discussion

### 3.1. Theoretical Calculations

**Figure 3:**
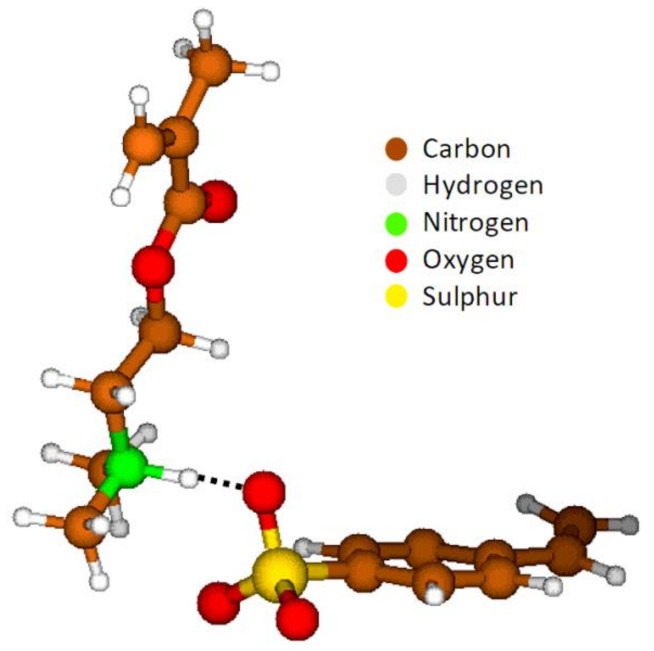
Geometrical feature of NaSS and DMAEMA ionic monomers in water.

Before studying the formation of the ionic network in the polymer film, one important point was to ensure that the interactions between the opposite charges of NaSS and DMAEMA were energetically favored over their corresponding counterions. Hence, DFT calculations were performed to study the interactions between the NaSS and DMAEMA ionic monomers in terms of binding energy. The estimated binding energy of ΔE = −109.16 kcal/mol is ascribed to the ionic interaction between the negatively charged sulfonate moiety of NaSS and the positively charged amino moiety of DMAEMA, reinforced by a H-bonding interaction established between the protonated DMAEMA and one oxygen atom of NaSS. The hydrogen bond shows a short H···O bond (1.361 Å) and an almost planar N-H···O angle (177°), see Figure 3, which suggests a rather strong interaction. This result indicates that the ionic complexation is feasible in selected ionic monomers systems.

### 3.2. Characteristics of Anionically and Cationically Charged Latexes

The charged latexes were synthesized by seeded semicontinuous emulsion polymerization in presence of 1–3% of ionic monomer. In case of NaSS, an emulsifier-free polymer latex was obtained, whereas in case of DMAEMA, non-ionic surfactant was used to improve the colloidal stability of the resulting latex.

**Figure 4:**
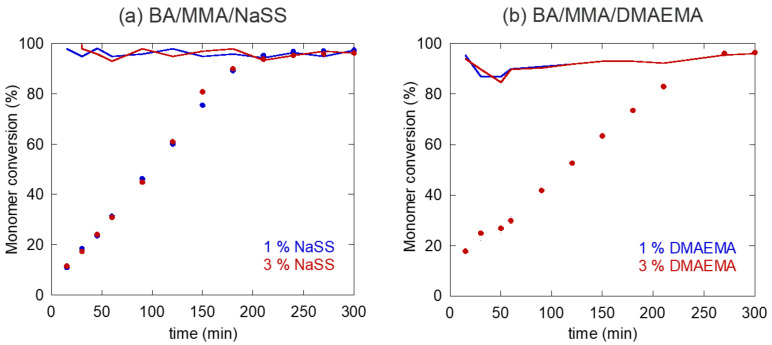
Monomer conversion evolution for latexes (**a**) containing NaSS and (**b**) containing DMAEMA. The continuous line represents the instantaneous conversion, while the dots the overall conversion.

The time evolution of monomer conversion (total and instantaneous) during the synthesis of both latexes in presence of different amount of ionic monomers is presented in Figure 4. Almost full conversion was achieved at the end of the polymerization process in all cases. The instantaneous conversions were high along with the reactions, indicating very low monomer concentration in the reactor during the syntheses. In both cases, no significant effect of ionic monomer concentration on the reaction kinetics was observed.

The main characteristics of the polymer latexes are presented in Table 2. Regarding the average particle size (d_p_), by increasing the content of ionic monomer from 1% to 3%, the average particle size increased for both anionic and cationic latexes. Similar behavior was reported for the case of NaSS [37], which occurs due to increasing of the ionic strength in the latex, screening the stabilization effect of the ionic groups incorporated into polymer particles.

It can be seen in Table 2 that the quantity of ionic species incorporated into polymer particles increased with the ionic monomers concentration. According to Sevilay et al. [37], this effect was attributed to the increased ionic strength in the system that shifted the absorption equilibrium towards polymer particles, which also explains why no significant difference on quantity of oligoradicals in aqueous phase was observed with increasing ionic monomer concentration. NaSS was incorporated, importantly, more than the DMAEMA monomer, which is also in agreement with previous works reported using cationic monomers [43]. Furthermore, as it may be observed from Table 2, in case of the cationic monomer, a higher amount of water-soluble species were formed. It is worth mentioning that this amount may be slightly underestimated, due to the decreased solubility of oligomers containing DMAEMA units at neutral pH, at which the phases were separated during the procedure of determination of water-soluble species.

For both latexes, the incorporated quantity of ionic monomers increased with their concentration, and consequently the surface charge density increased as well. The effect is more pronounced for NaSS.

The polymer microstructure was analyzed by means of the determination of the insoluble part of the polymer in THF (gel content) and the molar mass of the soluble part of the polymer. As observed in Table 3 the gel content for the anionically charged polymers was above 50%, while it was above 30% when DMAEMA was employed. The high values obtained could be attributed to the presence of ionic species, which are not soluble in organic solvents since in seeded semibatch emulsion polymerizations the formation of crosslinked structures in MMA/BA systems is almost negligible (<5 wt%) [49]. Nevertheless, there might be a contribution of branching and crosslinking reactions in these latexes since it is well known that TBHP initiator is efficient in hydrogen abstraction [37]. The soluble fraction of the polymer was analyzed by SEC and the measured molar masses are shown in Table 3. The molar masses of the anionic latexes were lower than the cationic ones due to the larger amount of gel content, in which the larger molar masses were incorporated (Table 3). The polydispersity index values are in the range of the ones normally obtained in the MMA/BA emulsion polymerizations.

### 3.3. Performance of the Polymer Films

Polymer particles functionalized with 1% and 3% of NaSS and DMAEMA were blended at two different pHs. In the first set of blends, the ionic interactions between the particles were promoted by keeping the pH below 7.5, at which, both types of polymer particles were charged. The second set of blends was prepared at pH above the pKa of the poly(DMAEMA) (pH > 7.5), which ensures its neutral state, avoiding ionic interactions between the particles. The blends obtained at pH > 7.5 were considered as reference material. Furthermore, the blending process was based either on the same surface charge density (Blends C1-1, C3-3 and C1-3) or on the same number of particles (Blends P1-1, P3-3 and P1-3) in both latexes. The films were prepared from the blended latexes. As shown in Figures S1 and S2 in the Supporting Information, homogeneous and transparent films were obtained in all the cases.

Similar T_g_ values of the reference and ionic complex materials were observed in a range of 18–20 °C, indicating that there is no important effect of the ionic complexation on the glass transition of the resulting polymer blends, probably because the main monomers in the blended polymers were the same (MMA/BA in 50/50 wt. ratio).

Recently, the relation between the surface charge density and surface wetting properties of polymer film was demonstrated in plasma-treated polymer films, that recovered the hydrophobicity due to a decrease of surface charge density [50]. This means that in case of ionic complexation, the neutralization of the surface charges within the polymer films might turn the surface more hydrophobic. Therefore, the change of the water contact angles (WCA) of the ionic complexed films with respect to reference film can be a solid suggestion of the established ionic complexes. In Table 4, WCA of the ionic complexes and reference films is shown. Lower WCA for reference films than for the ionic complexes was observed in all cases, probably owing to the presence of free ionic species that increase the hydrophilicity of the reference polymer films. After establishing ionic interactions, the higher WCA suggests hydrophobicity of the films.

**Figure 5:**
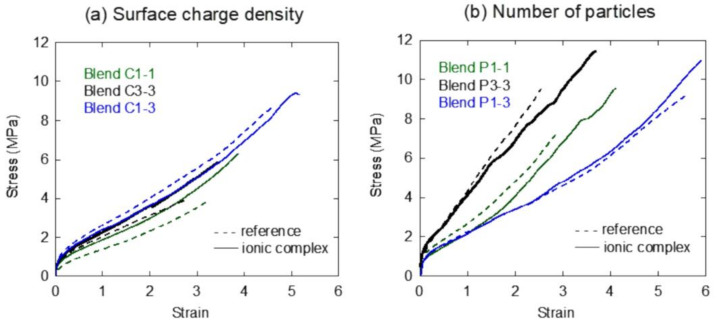
Mechanical properties measured at macroscopic level between oppositely charged polymer particles blends performed based on equal (**a**) surface charge density and (**b**) number of particles and at different pH. The dash lines represent the reference latex (pH > 7.5) while the continuous lines the ionic complex (pH < 7.5).

The mechanical properties of the blend materials, evaluated by the tensile test, are presented in Figure 5 and in Tables S3 and S4 in Supporting Information. In Figure S3, the stress–strain curves of the functional polymers with 1% and 3% NaSS and DMAEMA are shown. Anionically charged polymers with NaSS produced much more mechanically resistant films, with higher Young modulus and lower elongation at break than the cationically charged films produced with DMAEMA. In case of increased functional monomer quantity, slightly enhanced properties can be observed, especially the eongation at break, resulting in much more flexible films.

In Figure 5a, where stress–strain curves of Blends C1-1, C3-3 and C1-3 are presented, slightly higher Young’s modules, ultimate strength and toughness (MPa), with a clearly higher elongation at break (Table S3, Supporting Information) were obtained for the ionic complexed systems, which may be directly related to the ionic bonding effect. This effect is similar for Blends C1-1 and C3-3, indicating that the higher surface density in the last one did not affect additionally the ionic complexation process and the properties of the blend film. Probably this is a consequence of the addition of higher amount of cationic latex, due to the lower incorporation of cationic monomer and lower surface charge density, Table 2 in both cases. As there will be much more cationic than anionic particles in the latex blend, there is a space limitation to the complexation process. It is worth mentioning that the ionic bonding was established during the film formation, where agitation is avoided and particles containing DMAEMA likely tend to group together due to the larger number. According to Table 2, where the surface charge density of the different particles is shown, it can be observed that 1% NaSS latex presents similar charge density with that of 3% DMAEMA latex, along with similar particle sizes: 275 nm, 17 µC/cm^2^ and 250 nm, 19 µC/cm^2^, respectively. Therefore, to produce Blend C1-3, these two latexes were blended. According to Figure 5a, there is no improvement effect observed for the ionic complex. The slightly better properties of C1-3 with respect to C1-1 and C3-3 blends are likely result on the higher content of NaSS latex (Table 1), which presents mechanically more resistant films (Figure S3).

A similar effect of ionic bonding was observed for Blends P1-1, P3-3 and P1-3 (Figure 5b), in terms of slightly higher ultimate strength, toughness (MPa) and elongation at break values (Table S4, Supporting Information). However, a small drop in Young modulus was observed in this set of the blends after the complexation. This effect is stronger for Blend P3-3 than for Blend P1-1, probably due to the higher surface charge density of the blended particles (Table 2). Even though similar number of charged particles was incorporated into the blends, likely due to their difference in the surface charge density, the observed effects are rather modest. Nevertheless, the tensile characteristic of the Blend P1-3, prepared by employing 1 % NaSS latex (275 nm, 16 µC/cm^2^) and 3 % DMAEMA latex (250 nm, 19 µC/cm^2^), which allow blending of similar particle number with similar particle charge, again resulted in modest improvement of the properties (Table S4, Supporting Information).

**Figure 6:**
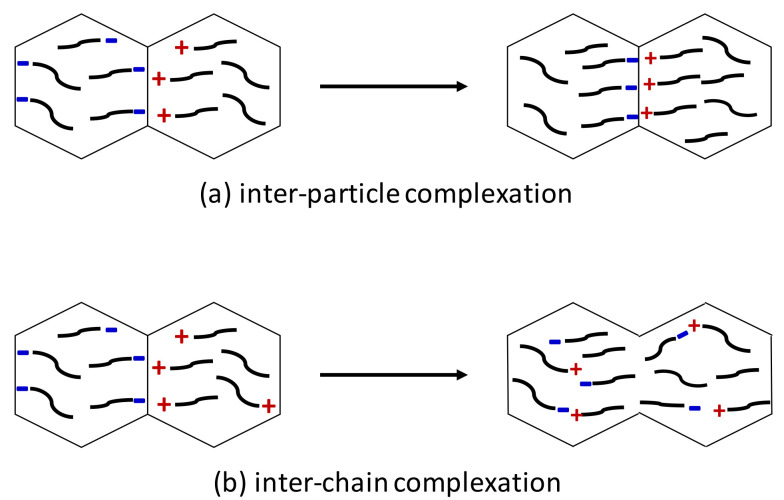
Schematic view of ionic complexation step at (**a**) inter-particle level and (**b**) inter-chain level.

Taking into consideration the particle systems blended in this work, ionic complexation might occur at two levels. When particles with opposite charges approach each other during film formation, the ionic complexation might occur either in the first step of particle packaging (inter-particle complexation, Figure 6a) or in the second step when the chain interdiffusion occur (inter-chain complexation, Figure 6b). In the former case, the formation of inter-particle ionic complexes would prevent the chain interdiffusion.

Few techniques have been developed to quantitatively measure the polymer chain diffusion in latex films. The most actively used methods are Small-Angle Neutron Scattering (SANS) and Fluorescence Resonance Energy Transfer (FRET) [51]. Although the SANS technique is appropriate for measuring the interdiffusion over distances comparable with the particle size, it is not adequate for measuring at shorter distances, hence, in this work FRET technique was chosen [52,53]. For that aim, dye-labeled charged polymer latexes were prepared with similar properties (conversion, particle size, incorporation, surface density and molecular weight) to the unlabeled polymer latex. Details of synthesis can be found in Supporting Information (Table S5).

**Figure 7:**
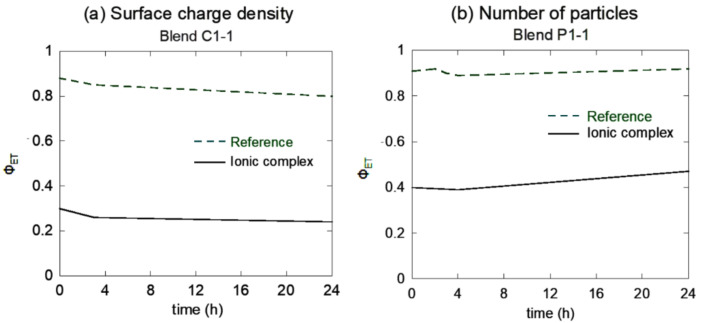
Evolution of quantum efficiency energy transfer during blending of oppositely charged polymer particles blends, performed at different pH for (**a**) Blend C1-1, based on the same surface charge density; and (**b**) Blend P1-1, based on the same number of particles.

The influence of the complexation process on the polymer chain diffusion was examined following the extent of energy transfer in newly formed polymer films from the blends of 1% NaSS and 1% DMAEMA (Blend C1-1 and Blend P1-1). Figure 7 compares the evolution of quantum efficiency energy transfer (Φ_ET_) over time for both polymer films, ionic complex (pH < 7.5) and reference material (pH > 7.5). As observed, in both cases studied (Figure 7a,b), Φ_ET_ values were maintained almost constant throughout the time, being much lower for the ionic complexed material.

These results show that at the present drying conditions, in case of ionic complexed material (pH < 7.5), the interdiffusion of polymer chains between neighboring particles was significantly lower. This effect is attributed to the presence of inter-particles ionic bonds, which created a network of bonded polymer chains rich in ionic monomer units. This ionic network conveys flexibility to the complexed films, as observed in tensile tests results. However, it simultaneously prevents the chain interdiffusion, accounted for the modestly enhanced mechanical properties.

By comparison of Figure 7a,b, one may observe that the blend prepared with equal particle sizes (P1-1) present higher chain interdiffusion degree than the blend with equal surface charge densities (C1-1). This is an indication of less ionic complexes established in case of P1-1, which clearly explain the lowest effect on mechanical properties, observed in Figure 5.

For practical application of waterborne polymer coatings, their sensitivity to water is an essential characteristic. Usually, the presence of ionic species within the polymer films, from either the surfactant, ionic monomers or other components, increases their water sensitivity. For instance, it was shown that hydrophilic block copolymers containing AA in the shell, presented high water sensitivity [53]. In the work of Tiggelman and coworkers [31], the main drawback of the material formed by ionic complexation of AA containing particles and DMAEMA ones, was the high water uptake (more than 15%). However, Sevilay et al. have demonstrated that, in case of NaSS stabilized polymer films, this increment occurred sharply in the initial contact with water, after which the water uptake remains constant and usually much lower than the film in which conventional surfactant was employed [34]. The high initial water uptake occurred due to the anionic network formed within the film, which leads to saturation, whereas in the case of conventional surfactants, they formed a hydrophilic pocket able to absorb much larger water quantities.

**Figure 8:**
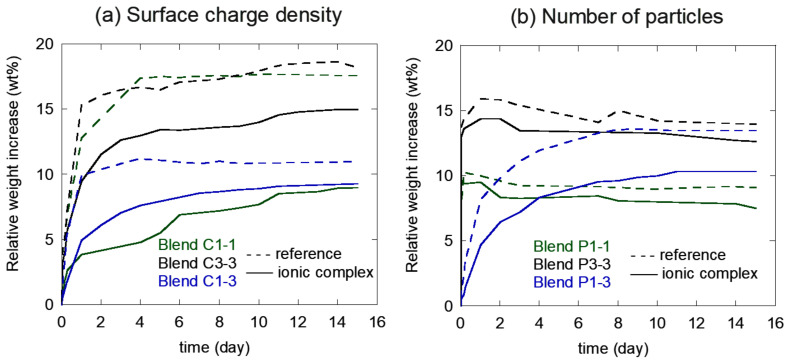
Evolution of water uptake by the blend films (**a**) based on equal surface charge density; and (**b**) based on equal number of particles.

The water sensitivity of the blends prepared in this study, measured by means of water uptake, is presented in Figure 8.

As it can be seen in Figure 8a, the reference Blends C1-1 and C3-3 present similar behavior absorbing up to almost 20 wt% of water, likely due to high sulfonate ions concentration (at pH of preparation of reference blends, the cationic latex is mostly in neutral state). Conversely, the ionic complex Blends C1-1 and C3-3 presented much lower water uptake, which moreover depends on the surface charge density. Therefore, ionic complex C1-1 absorbs about 8 wt%, importantly less than C3-3 (14 wt%). The drop of water uptake with respect to reference blends is likely due to the ionic complexation process that led to the neutralization of most of the present charges, pointing out that very few free charges are presented in the complex films. In ionic complex C3-3 there is still a high number of free charges, probably due to the mentioned steric hindrance to the complexation reaction. The same reason is behind the observed slightly higher water uptake of ionic complex C1-3 than the ionic complex C1-1.

The water uptake of the polymer films prepared by blending the same number of particles is shown in Figure 8b. The reference and ionic complex Blends P1-1 and P3-3 showed similar water sensitivity during the first hours, being high the amount of water that penetrated within the film (10 wt% and 15 wt%, respectively). Relatively smaller difference between the reference and ionic complex blends is a consequence of the excess free sulfonate charges (the surface charge density of the anionic particles was higher, Table 2). However, the water absorption by ionic complex Blends P1-1 and P3-3 dropped after the first two hours, probably owing to the weight loss of the polymer film. Some water-soluble species were dissolved by the penetrating water and desorbed from the film to the water phase. For the blend with much lower difference in density charge, Blend P1-3, clearly, the ionic complex material did not absorb as much water as the reference one during the first hours, probably due to a higher degree of neutralization in this case and less amount of free charges.

**Figure 9:**
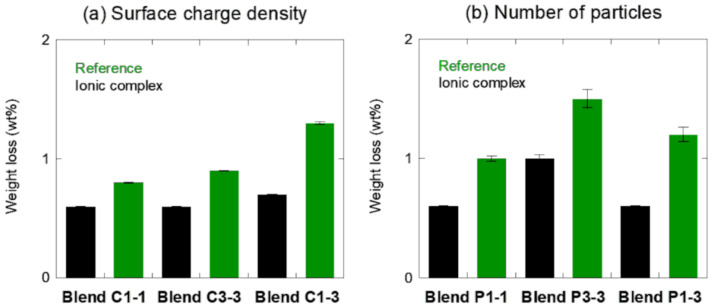
Weight loss (wt%) values after water uptake experiments for polymer blend films based on equal (**a**) surface charge density; and (**b**) number of particles.

In addition, films were dried and weighted before and after their immersion in water to determine the weight loss of the materials. The weight loss (wt%) is referred to the initial weight of the samples (Figure 9).

All the films were prepared using the same latexes but the pH was altered. It can be seen that in all the cases the reference films contained higher amount of soluble polymer chains. Although dialyzed latexes were used for these blends, polymer chains with higher molecular weight than the membrane cut-off (12,000–14,000 Da) were not eliminated, contributing to the observed weight loss as they may have migrated from the film. It can be said that for Blends C1-1, C3-3 and C1-3, the amount of material lost was small (<1 wt%, <1 wt% and <1.5 wt%, respectively), whereas for Blend P1-1, P3-3 and P1-3, the lost was slightly higher (<1 wt%, <1.5 wt% and <1.5 wt%, respectively). The difference between the reference film with respect to the ionic complex might be due to a decreased desorption of water-soluble chains from film to water phase, prevented by the presence of ionic complexes, actuating as physical barriers. The highest difference was observed for ionic complex C1-3 and P1-3, indicating that the extent of ionic bonds for these blends were higher than for the others, due to the decrease of steric limitations to the ionic bonding reactions.

## 4. Conclusions

In this work, ionic complexation between waterborne particles and its effect on the final film performance was studied. The oppositely charged polymer particles were prepared by emulsion polymerization of MMA/BA main monomers in presence of small amount (1–3%) of either NaSS or DMAEMA ionic monomers. Density functional theory calculations showed that the interaction between main ions of NaSS and DMAEMA was favored against the interaction with their respective counter ions, providing a fundamental background on the formation of an ionic complexation when mixing oppositely charged polymeric dispersions. The blends were prepared varying two parameters: surface charge density and number of particles. As pKa of DMAEMA is about 7.5, all the blends were prepared at two different pHs. At pH< 7.5 ionic interactions were expected, and ionic complexed films were prepared, and at pH > 7.5 no ionic interactions happen, allowing the preparation of reference films.

Mechanical properties tested by tensile measurements showed that the ionic complexed blends, presented slightly better mechanical properties than the reference blend, although the effect was rather modest in all studied combinations. The modest result obtained was attributed to the steric limitation that individual particles have during film formation process, due to differences in number of particles and surface charge densities, along with a lack of agitation. In such conditions, many charges remain free. Nevertheless, the modest enhancement of mechanical properties was kept even in the blends prepared with similar particle size and surface charge density.

Considering these results, FRET technique was used to examine if the ionic bonding occurs at molecular or particle level during blending at both pHs. The results revealed that in the ionic complexed blends, the interdiffusion of polymer chains between neighboring particles was hindered with respect to the reference blend. It was thought that the created network of ionic complexed polymer chains shell around individual polymer particles within the film decreased the overall level of chain interdiffusion, however, it slightly reinforced the film. The reference blend presented enhanced polymer chain mobility, but lower mechanical performance. This means that when ionic network is formed, polymer chains diffusion is affected, and consequently slight mechanical properties improvement is observed.

Conversely, the reference blends prepared at pH > 7.5 clearly showed higher water absorption compared to the ionic complex blend (pH < 7.5), owing to the lack of neutralization between ionic species in the former. Exception are the films prepared by blending based on the same particle number (P1-1 and P3-3), where the greater surface charge density of NaSS particles is responsible for such high water penetration within blend materials, screening the possible effect of formed ionic interaction. Despite that, dialyzed latexes were used throughout this study, and a migration of polymer chains with higher molecular weight than the membrane cut-off (14,000 Da) might contribute to the observed weight loss from the polymer blends during water uptake measurements.

The presented inter-particle complex between sulfonate–amine groups containing latexes open a promising approach for reinforcing polymer films cast from water-based polymers. However, this concept still needs further investigation in order to reinforce the established interactions.

## Figures and Tables

**Table 1:** Receipts for polymer blends preparation.

	Surface Charge Density (µC/cm^2^)			Number of Particles (Np/L)		
Latex	Blend C1-1	Blend C3-3	Blend C1-3	Blend P1-1	Blend P3-3	Blend P1-3
1% NaSS	3 mL	-	5 mL	5 mL	-	5 mL
3% NaSS	-	3 mL	-	-	5 mL	-
1% DMAEMA	5 mL	-	-	4 mL	-	-
3% DMAEMA	-	5 mL	4 mL	-	3 mL	4 mL

**Table 2:** Average particle size, surface incorporation of ionic monomer and water-soluble species measurements for anionically and cationically charged latex.

Latex	d_p_ (nm)	Incorporation (% Ionic Monomer)	Surface Charge Density (µC/cm^2^)	Water-Soluble Species (% Ionic Monomer)
1% NaSS	275 ± 5	70 ± 6	16 ± 2	30 ± 2
3% NaSS	300 ± 4	52 ± 3	36 ± 4	35 ± 3
1% DMAEMA	240 ± 5	33 ± 4	9 ± 2	42 ± 10
3% DMAEMA	250 ± 2	19 ± 4	19 ± 3	45 ± 10

**Table 3:** Microstructure of the charged polymers.

Latex	Insoluble Polymer (gel) (wt%)	M_w_ (KDa)	Đ
1% NaSS	53 ± 2	304	2.5
3% NaSS	55 ± 1	260	2.4
1% DMAEMA	40 ± 2	350	2.0
3% DMAEMA	30 ± 1	340	2.1

**Table 4:** Contact angle measurements for reference and ionic complex films.

Blend System	WCA (°)
Blend C1-1, reference	64 ± 4
Blend C1-1, ionic complex	90 ± 3
Blend C3-3, reference	64 ± 2
Blend C3-3, ionic complex	87 ± 3
Blend C1-3, reference	76 ± 2
Blend C1-3, ionic complex	90 ± 1
Blend P1-1, reference	78 ± 1
Blend P1-1, ionic complex	87 ± 2
Blend P3-3, reference	86 ± 3
Blend P3-3, ionic complex	94 ± 4
Blend P1-3, reference	74 ± 1
Blend P1-3, ionic complex	91 ± 1

## Data Availability

The data presented in this study are available on request from the corresponding author.

## Supplementary Materials

The following are available online at https://www.mdpi.com/article/10.3390/polym13183098/s1; Figure S1: Appearance of the polymer blend films obtained based on equal surface charge density; Figure S2: Appearance of the polymer blend films obtained based on equal number of oppositely charged particles; Figure S3: Stress-strain curves for NaSS and DMAEMA original films. Table S1: Formulation used for anionically and cationically charged dispersion seed; Table S2: Formulation used for anionically and cationically charged polymer particles; Table S3: Mechanical properties of the polymer blends based on equal surface charge densities at different pH; Table S4: Mechanical properties of the polymer blends based on the same number of particles at different pH; Table S5: Characteristics of labeled dye anionically and cationically charged dispersions.
