# Supplementary material for: Ionic Inter-Particle Complexation Effect on the Performance of Waterborne Coatings
Source: Polymers (Basel). 2021 Sep 14;13(18):3098. doi: 10.3390/polym13183098 (PMC8470605; doi:10.3390/polym13183098)
Supplement: Supplementary file 1 [file polymers-13-03098-s001.zip › polymers-1382791-supplementary.pdf]

SUPPORTING INFORMATION

Electrostatic inter-particle complexation effect on the performance of waterborne coatings

Maialen Argaiz[1], Fernando Ruipérez[2], Miren Aguirre[1] and Radmila Tomovska[1,3]

[1]POLYMAT andDepartamento de Química Aplicada, Facultad de Ciencias Químicas, University of the Basque Country UPV/EHU, Joxe Mari Korta zentroa, Tolosa Hiribidea 72, 20018 Donostia-San Sebastián, Spain
[2]POLYMAT and Physical Chemistry Department, Faculty of Pharmacy, University of the Basque Country UPV/EHU, 01006 Vitoria-Gasteiz, Spain
[3]Ikerbasque, Basque Foundation for science, Maria Diaz de Haro 3, 48013 Bilbao, 48013, Spain

1. Synthesis of waterborne polymer latexes. The seed formulation for anionically and cationically charged polymer particles is represented in Table S1. The seeds were synthetized as follows. The initial charge, the acrylic monomers MMA and BA together with the functional monomer (F.M.) (2 wbm% and 3 wbm% for anionic and cationic dispersion, respectively) and the emulsifier (4 wbm%) in the case of DMAEMA was charged into 1 jacketed glass reactors. The reactor was equipped with reflux condenser, N_2 inlet, temperature probe, 3 feeding inlets, sample device and stainless steel 4 frame type stirrer. The feeding flow rates and the temperature were controlled by an automatic control system (Camile TG). Firstly, the initial charge was mixed for 20 minutes under N_2 at 200 rpm. Then, the reaction temperature was increased to the desire one, 70 ^{o}C and 60 ^{o}C for anionic and cationic dispersions, respectively. Feeding of the initiator system was started in both cases, together with the feeding of monomer system in case of the cationic dispersion.

Table S1. Formulation used for anionically and cationically charge dispersion seed.

Compounds (wt%)	Initial charge		Stream 1		Stream 2		Stream 3	
	NaSS	DMAEMA	NaSS	DMAEMA	NaSS	DMAEMA	NaSS	DMAEMA
**MMA**	5	-	-	10	-	-	-	-
**BA**	5	-	-	10	-	-	-	-
**F.M**	2*	-		-	-		-	3*
**Disponil AFX2075**	-	4*	-	-	-	-	-	-
**TBHP**	-	-	0.5*	-	-	1*	-	-
**AsAc**	-	-		-	0.5*	-	-	1*
**H_2O**	70	60	10	-	10	10	-	10

*weight based on main monomers (BA/MMA) (wbm%)

The formulations for the seeded semibatch anionically and cationically charged polymer particles is represented in Table S2.

Table S2. Formulation used for anionically and cationically charge polymer particles.

Compounds (wt%)	Initial charge		Stream 1		Stream 2		Stream 3	
	NaSS	DMAEMA	NaSS	DMAEMA	NaSS	DMAEMA	NaSS	DMAEMA
**Seed**	39	44	-	-	-	-	-	-
**MMA**	-	-	23	20	-	-	-	-
**BA**	-	-	23	20	-	-	-	-
**F.M**	-	-	-	-	-	-	1-3*	1-3*
**Disponil AFX2075**	-	-	-	-	-	-	-	4*
**TBHP**	-	-	-	-	0.5*	0.2*	-	0.2*
**AsAc**	-	-	-	-	-	-	0.5*	-
**H_2O**	-	-	-	-	5	8	10	8

*weight based on main monomers (BA/MMA) (wbm%)

2. Synthesis of polymer latexes for FRET analysis Over the past 20 years, the group of Prof. Winnik at University of Toronto have used FRET technique to study a variety of factors that affect the rate of polymer diffusion in latex films[1,2]. FRET is referred to non-radiative transmission of energy from a donor molecule to an acceptor molecule. The donor molecule is the dye or chromophore that initially absorbs the energy and the acceptor is the chromophore to which the energy is subsequently transfer[3,4]. Hence, the energy that is captured by the donor upon its excitation is transferred to the acceptor. Although there are large different parameters that can affect FRET experiments, the distance between donor/acceptor molecules is an important one.

The basic principle of this technique is that when polymer dispersions containing donor and acceptor labelled polymer particles are dried to form a film, donor and acceptor labelled polymer chains are separated due to boundaries between particles. Once these polymer chains diffuse across the boundaries, they bring donor and acceptor dyes into proximity, allowing the extent of energy transfer to increase and leading to a faster decrease in the fluorescence decay profile. Following this evolution, the quantum efficiency energy transfer (ϕ_{ET}) was calculated

3. FRET data acquisition: Fluorescence decay profile were measured by Time Correlated Single Photon Counting (TCSPC) carried out using the Fluoromax-4 appartus (Horiba, Jobin-Yvon) equipped with a single photon counting controller (FluoroHub, Horiba Jobin-Yvon) and a

pulsed diode light source NanoLED emiting at 300 nm. Emission from the sample was detected at 360 nm. Each measurement was continued until 10000 counts were acquired in the maximum channel.

4. FRET data analysis. The last step of Film Formation process, where polymer chains from neighbor particles might diffuse was monitored by measuring the energy extent between donor and acceptor labelled particles. In this technique, donor fluorophore, initially in its electronic excited state, might transfer energy to any nearby acceptor fluorophore through non-radiative dipole-dipole coupling. As observed in equation 1, the rate of energy transfer (w (t)) strongly depends on the distance between the donor and acceptor fluorophores (sixth-power relationship)[5–7].

$$w\,(t) = \frac{1}{\tau_D^0}\left(\frac{R_{F0}}{r}\right)^6 \quad \textbf{(1)}$$

where τ_D^0 is the donor fluorescence lifetime in the absence of acceptors and R_{F0} refers to Föster distance at which energy transfer is 50 % efficient. Conceptually, the Föster critical distance is the maximal separation length between donor and acceptor labelled polymers under which energy transfer will still occur.

In the presence of isolated donor molecules, the fluorescence decay profile (I_D) is defined as the exponential decay function of time (t)[8].

$$I_D = Aexp\left(\frac{-t}{\tau_D^0}\right) \quad \textbf{(2)}$$

where A is a constant and τ_D^0 is the lifetime of the donor in absence of acceptor as explained above. τ_D^0 for Phe-BA/MMA/1 % NaSS labelled latex film was measured experimentally obtaining a value of 43.5 ns.

However, energy transfer for donors and acceptors randomly distributed in a three dimensional space, the donor fluorescence decay profile will have a stretch exponential form[7,8].

$$I_D\,(t) = \exp\left(-\frac{t}{\tau_D^0}\right)\exp\left[-2\delta\left(\frac{t}{\tau_D^0}\right)^{0.5}\right] \quad \textbf{(3)}$$

where δ is a constant parameter proportional to the concentration of acceptor and Föster distance.

In these experiments, each fluorescence decay profile was standardized to unit intensity and time zero (t_0). These values were fitted to the following equation[4,7,8]:

$$I_D\ (t) = \exp\left(-\frac{t}{\tau_D^0}\right)\exp\left[-2\delta\left(\frac{t}{\tau_D^0}\right)^{0.5}\right] + A_2\exp\left(-\frac{t}{\tau_D^0}\right) \quad \textbf{(4)}$$

where the first term corresponds to the region where donor and acceptor molecules are mixed, while the second term refers to the unmixed region where energy transfer does not occur. A_1 and A_2 parameters were obtained from the fitted of each decay profile, while fixed values for δ (0.45) and τ_D^0 (43.5 ns). The presented parameters were used to integrate I_D (t) analytically, from decay time zero to time infinity and to calculate the quantum efficiency energy transfer (Φ_{ET}).

The area under each standardized fluorescence decay profile is equal to the total fluorescence intensity from the sample, which is proportional to the quantum efficiency of fluorescence. One could calculate the quantum efficiency of energy transfer from the changes in the area under the decay profiles. The quantum efficiency of energy transfer (Φ_{ET}) is defined as follows[7,8].

$$\Phi_{ET}\ (t) = 1 - \frac{\int_0^\infty I_D\ (t)dt}{I_D^0\ (t)dt} \quad \textbf{(5)}$$

where $\int_0^\infty I_D\ (t)$ refers to the integrated area under the normalized decay profile and $I_D^0\ (t)$ is defined as the donor decay profile of the film containing donor fluorescence molecule (τ_D^0). τ_D^0 value was experimentally defined as 43.5 ns as explained above[8].

$$\Phi_{ET}\ (t) = 1 - \frac{\int_0^\infty I_D\ (t)dt}{\tau_D^0} = 1 - \frac{area\ (t)}{\tau_D^0} \quad \textbf{(6)}$$

5. Performance of polymer films**.** The appearance of the blended polymer films considering surface charge density and number of particles parameters is shown in Figure S1 and Figure S2, respectively.

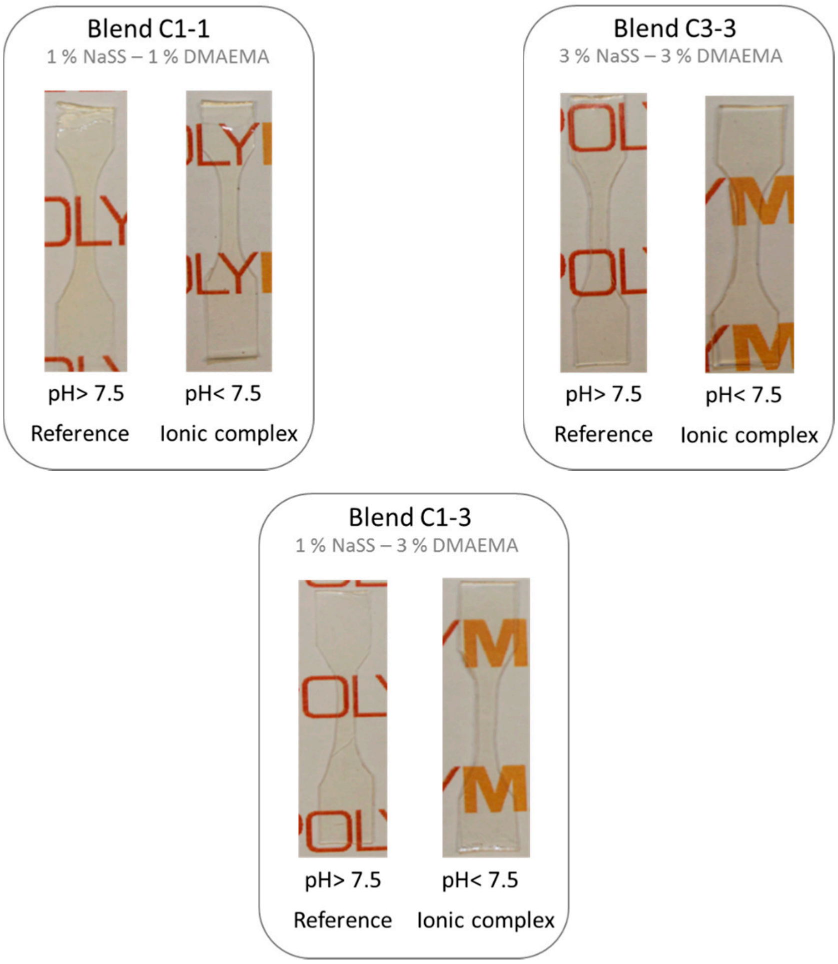

Figure S1. Appearance of the polymer blend films obtained based on equal number of opposite charges.

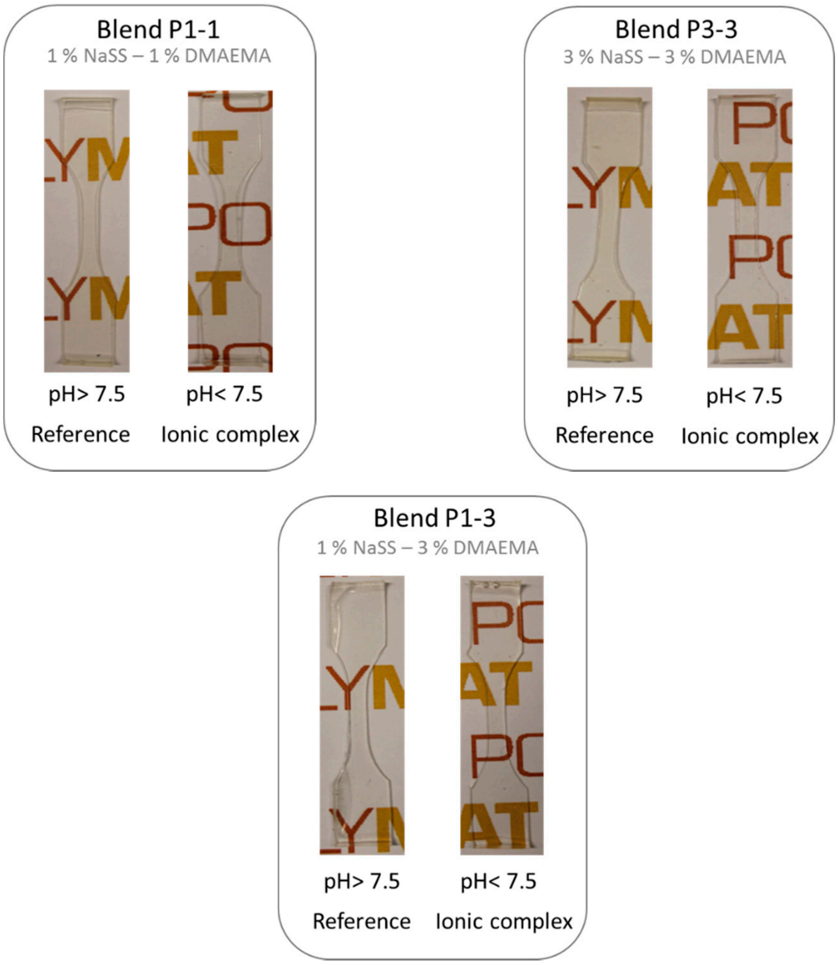

Figure S2. Appearance of the polymer blend films obtained based on equal number of opposite particles.

The values obtained for stress-strain plots parameters (Young modulus, elongation at break, ultimate strength and toughness) are presented in Table S3 and Table S4.

Table S3. Mechanical properties of the polymer blends performed at equal surface charge densities and different pH, related to the stress-strain plots represented in Figure 6.

Blend	Young´s modulus (MPa)	Elongation at break	Ultimate strength (MPa)	Toughness (MPa)
C1-1, reference	3 ± 2	3.7 ± 1.0	4.5 ± 1.1	7.1 ± 1.1
C1-1, ionic complex	4 ± 1	3.8 ± 1.1	6.1 ± 0.6	12.1 ± 1.6
C3-3, reference	5 ± 1	3.1 ± 0.7	3.9 ± 0.2	7.1 ± 1.8
C3-3, ionic complex	6 ± 2	3.5 ± 0.4	5.8 ± 0.4	11.3 ± 1.7
C1-3, reference	5 ± 2	4.6 ± 0.9	8.7 ± 0.5	21.5 ± 1.7
C1-3, ionic complex	5 ± 1	5.2 ± 1.0	9.5 ± 1.3	24.4 ± 1.5

Table S4. Mechanical properties of the polymer blends based on the same number of particles at different pH, related to the stress-strain plots represented in Figure 7.

Blend	Young´s modulus (MPa)	Elongation at break	Ultimate strength (MPa)	Toughness (MPa)
P1-1, reference	7 ± 2	3.1 ± 0.3	7.4 ± 0.7	11.9 ± 2.8
P1-1, ionic complex	6 ± 1	3.8 ± 0.5	9.1 ± 2.3	18.5 ± 4.1
P3-3, reference	16 ± 3	2.4 ± 0.2	8.3 ± 1.2	12.1 ± 2.3
P3-3, ionic complex	14 ± 2	3.7 ± 0.5	11.1 ± 1.4	22.8 ± 1.6
P1-3, reference	5 ± 1	5.4 ± 0.2	9.4 ± 0.5	25.1 ± 3.6
P1-3, ionic complex	4 ± 1	5.7 ± 0.2	10.9 ± 0.4	28.3 ± 1.6

The stress-strain curves for NaSS and DMAEMA containing polymer films is illustrated in Figure S3.

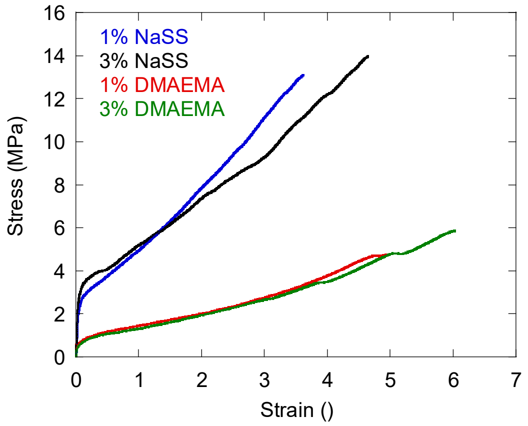

Figure S3. Stress-strain curves for NaSS and DMAEMA original films.

6. Characterization for dye labeled latexes. High conversion was achieved at the end of the polymerization processes. Anionically and cationically charged dispersions showed similar characteristics compared to unlabeled dye latex (Table 2 and Table 3).

Table S5. Characteristics of labeled dye anionically and cationically charged dispersions.

Latex/Characterization	1 % NaSS-FRET	1 % DMAEMA-FRET
Conversion (%)	97	93
dp (nm)	255 ± 5	245 ± 5
Incorporation (% F.M)	75 ± 2	21 ± 5
Surface charge density (μC/cm^2)	19 ± 2	6 ± 1
Insoluble polymer (%wt)	51 ± 1	40 ± 5
Mw (KDa)	280 ± 10	310 ± 25
Đ	2.5	1.6

References

1. Zhao, C.-L.; Wang, Y.; Hruska, Z.; Winnik, M.A. Molecular Aspects of Latex Film Formation: An Energy-Transfer Study. *Macromolecules* **1990**, *23*, 4082–4087, doi:10.1021/ma00220a009.

2. Wang, Y.; Zhao, C.; Winnik, M.A. Molecular diffusion and latex film formation: An analysis of direct nonradiative energy transfer experiments. *J. Chem. Phys.* **1991**, *95*, 2143–2153, doi:10.1063/1.461013.

3. McPhie, P. *Principles of Fluorescence Spectroscopy, Second ed. Joseph R. Lakowicz*; 2000; Vol. 287; ISBN 0387312781.

4. Wu, J.; Winnik, M.A.; Farwaha, R.; Rademacher, J. Effect of a Water-Soluble Polymer on Polymer Interdiffusion in P(MMA-co-BA) Latex Films. *Macromol. Chem. Phys.* **2003**, *204*, 1933–1940, doi:10.1002/macp.200350060.

5. Farinha, J.P.S.; Vorobyova, O.; Winnik, M.A. An energy tranfer study of the interface thickness in blends of poly(butyl methacrylate) and poly(2-ethylhexyl methacrylate). *Macromolecules* **2000**, *33*, 5863–5873, doi:10.1021/ma0000935.

6. Keddie, J..; Routh *Fundamentals of Latex Film Formation*; Springer laboratory: Dordrecht, Netherlands, 2010; ISBN 978-90-481-2844-0.

7. Kobayashi, M.; Rharbi, Y.; Winnik, M.A. Effect of inorganic pigments on polymer interdiffusion in a Low-Tg latex film. *Macromolecules* **2001**, *34*, 1855–1863, doi:10.1021/ma000604n.

8. Pinenq, P.; Winnik, M.A.; Ernst, B.; Juhué, D. Polymer diffusion and mechanical properties of films prepared from crosslinked latex particles. *J. Coatings Technol.* **2000**, *72*, 45–61, doi:10.1007/bf02697987.
